# Estimation of the air conditioning energy consumption of a classroom using machine learning in a tropical climate

**DOI:** 10.3389/fdata.2025.1520574

**Published:** 2025-05-14

**Authors:** Liliana Ortega-Diaz, Julian Jaramillo-Ibarra, German Osma-Pinto

**Affiliations:** ^1^Grupo de Investigación en Sistemas de Energía Eléctrica (GISEL), Universidad Industrial de Santander, Bucaramanga, Colombia; ^2^Grupo de Investigación en Energía y Medio Ambinete (GIEMA), Universidad Industrial de Santander, Bucaramanga, Colombia

**Keywords:** classroom, building, air conditioning, prediction, machine learning

## Abstract

Air conditioning energy consumption in buildings represents a considerable percentage of total energy consumption, which underlines the importance of implementing measures contributing to its reduction. Predicting energy consumption is critical to making informed decisions and identifying factors influencing power consumption. Machine learning is the most widely used approach for prediction due to its speed, accuracy, and non-linear modeling. In this study, three machine learning models were used to predict the air conditioning energy demand in a classroom of an educational building in a hot tropical climate. The models selected are SVR (Support Vector Regressor), DT (Decision Tree), and RFR (Random Forest Regressor) due to their wide use in the literature; therefore, the goal is to establish which one offers the best performance for this case study based on a comparative analysis using performance metrics. Cross-validation was used to perform robust training. Twenty-two input variables were considered: climatological, operational, and temporal. Occupancy is the variable with the highest correlation with air conditioning consumption; these two variables have a positive relationship of 0.65. Monitoring was carried out for 72 days, including weekends. Six study scenarios were considered, in which the monitoring period varied, influencing the number of samples. In addition, two sensitivity analyses were performed by modifying the time interval of the data (1, 5, 10, 20, 30, and 60 min) and the data split (50:50, 60:40, 70:30, 80:20 and 90:10). The evaluation of the models was performed using RMSE, MAE and *R*^2^ metrics, to different characteristics and approaches to error measurement. During the training phase, the RFR model achieved a coefficient of determination (*R*^2^) of 0.97, while the SVR obtained an *R*^2^ of 0.78 in the test phase. Finally, it is concluded that using shorter time intervals (every 1 min) in the data improves the performance of the predictive models. Splitting the data into 80:20 and 90:10 ratios resulted in the lowest RMSE values for the three models evaluated. Training the models with a larger amount of data allows for capturing more representative patterns, which improves their generalization ability and performance on new data.

## 1 Introduction

Globally, heating, ventilation, and air conditioning (HVAC) systems can account for up to 40% of total energy consumption in buildings (residential, commercial, educational, and industrial) (Kaushik and Naik, 2024; Gao et al., 2019). This consumption could double by 2050 if no action is taken; however, implementing passive designs, promoting behavioral changes, and using more efficient equipment can help reduce demand and alleviate pressure on electricity systems (Agency, 2023). Climate change, population and income growth, and thermal comfort preferences increase cooling consumption (Santamouris, 2016; Tocchio et al., 2024).

Accurate energy consumption prediction helps improve resource efficiency and promote sustainability (Zhang and Pei, 2024). In addition, this prediction is critical for improving air conditioning (AC) system operating efficiency and electricity demand response (Huang et al., 2024; Yang et al., 2024). It is possible to optimize the performance of cooling systems through optimal control, maximizing their energy efficiency and improving their performance under various operating conditions. This strategy allows dynamically adjusting system parameters, achieving more precise temperature regulation, reducing energy consumption, and extending equipment life (Xue et al., 2024). Studies have been conducted to reduce energy consumption without affecting thermal comfort by ensuring a balance between energy management and indoor environmental quality (Ogundiran et al., 2024).

Forecasting estimates energy consumption for a specific time in the future. The literature highlights four main approaches for this forecasting type: statistical methods, white box, black box, and gray box models. Each approach has advantages and disadvantages, which require the application of criteria to choose the most appropriate one according to the case study, the prediction horizon, and the type of data available, among other factors. In recent years, machine learning models, also known as black box models, such as Artificial Neural Networks (ANN), Recurrent Neural Networks (RNN), Deep Neural Networks (DNN), Multi-Layer Perceptron (MLP), Decision Trees (DT), Multiple Linear Regression (MLR), Linear Regression (LR), Support Vector Machine Regression (SVR), Random Forests (RF), Gradient Boosting eXtreme (XGB), etc. have been preferred by researchers due to their speed and accuracy. These models have shown remarkable improvements in predicting energy consumption in buildings, especially in residential, commercial, and educational sectors, facilitating energy management, and implementing energy-saving strategies (Ortega-Diaz et al., 2023).

Recently, studies have been conducted on predicting cooling energy demand using machine learning techniques (Saeideh et al., 2024). An example is using machine learning and simulation to explain air conditioning in buildings (Duhirwe et al., 2024). Vergés et al. (2024) used neural networks to evaluate the energy implications of HVAC systems in nursing homes during the cooling season. Their study, which was based on data from eight nursing homes, showed excellent predictive ability (*R*^2^ = 0.95) and highlighted that adaptively adjusting operating temperatures can achieve energy savings of up to 23.4%. This work is especially relevant in warmer and drier climates, where energy efficiency is crucial for the comfort and wellbeing of residents. A model was developed to estimate the cooling capacity of air conditioners in real time using non-intrusive, low-cost, scalable parameters measured from the indoor unit. These parameters include three refrigerant pipe temperatures, compressor power, and other powers, which allow the capture of operating conditions and improve the model's accuracy. The artificial neural network processes this data to identify patterns and accurately estimate system performance (Sholahudin et al., 2024). Sundaram et al. (2024) introduced the use of LSTM networks to predict energy consumption in residential buildings with HVAC systems in the design phase, demonstrating superior accuracy (*R*^2^ = 0.97) and higher training efficiency (2.69 s for more than 500 test cases) compared to DNN and ANN models. This advance enables accurate estimates before construction and facilitates energy efficiency improvements from the earliest design stages. Liu et al. (2024) conducted a comparative analysis of various models for predicting air conditioner energy consumption across different periods in large public buildings. Additionally, techniques such as Support Vector Machines (SVMs), Artificial Neural Networks (ANNs), Narrow Neural Networks (NNN), and optimizable SVMs have been employed to forecast electricity usage in office buildings equipped with active chilled beam systems (Hajimirza Amin et al., 2024).

In contrast to other studies, some similarities and differences are found. For example, Saeideh et al. (2024) used only 768 samples of 9 physical variables to predict cooling consumption in residential buildings. In contrast, our study incorporates a broader set of variables influencing energy consumption and a more significant amount of data. The variables they considered are related to orientation, glazing area, relative compactness, total height, and surface area of the buildings. In other words, they considered physical variables not considered in this study since we are dealing with a single building, and these variables are constant over time. For this reason, the variables analyzed in this study are mainly climatological, operational, and occupancy variables. The accuracy obtained by Havaeji et al. was 97.4%, which is comparable to the 95% accuracy achieved in this study. The strength of our research lies in its ability to monitor various classroom characteristics over an extended period, capturing different scenarios that occur over several days. Additionally, although the buildings studied have other uses, the SVR model demonstrated an adequate ability to predict future values. Alawi et al. (2024) used the same data set as (Saeideh et al., 2024) generated through simulation. They used SVR and RF and split the data in a 70:30 ratio. However, they did not evaluate the impact of varying this data distribution. They took eight input variables for prediction and then presented various scenarios by performing input combinations. In another study, it was observed that the shortest time interval (10 min) provided the best performance, which aligns with the findings of this research. The models performed best using data with reduced time intervals (Liu et al., 2024). Another study used synthetic data from office buildings to predict cooling system consumption. They also trained the SVR and obtained an *R*^2^ of 0.95 as we did (Hajimirza Amin et al., 2024). Simulation-generated data require less effort and usually represent ideal conditions. In contrast, data collected through sensors and monitoring equipment involve more work but provide a more accurate and realistic representation of observed conditions.

Although several studies have been conducted on predicting energy consumption in buildings, there is a lack of research on the behavior of air conditioning consumption in classrooms. It is necessary to carry out studies of the interior spaces of a building to have a detailed analysis of its behavior. In this context, this study aims to evaluate the performance of three machine learning models to predict AC consumption in a classroom. The performance is evaluated using RMSE, MAE, and *R*^2^ metrics.

## 2 Methodology

### 2.1 Case study

**Figure 1:**
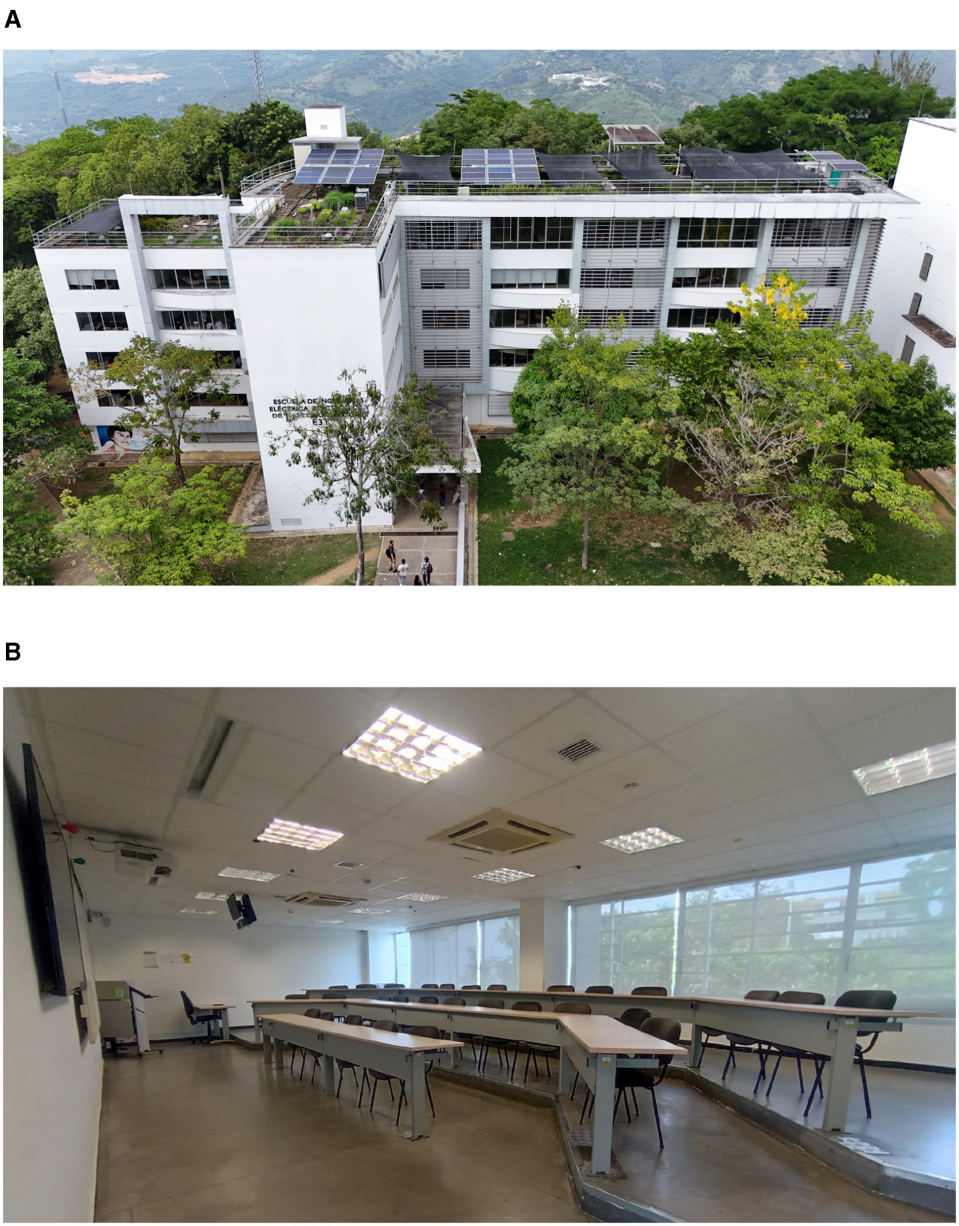
Case study. **(A)** Electrical, electronic and telecommunications engineering building. **(B)** Classroom.

**Figure 2:**
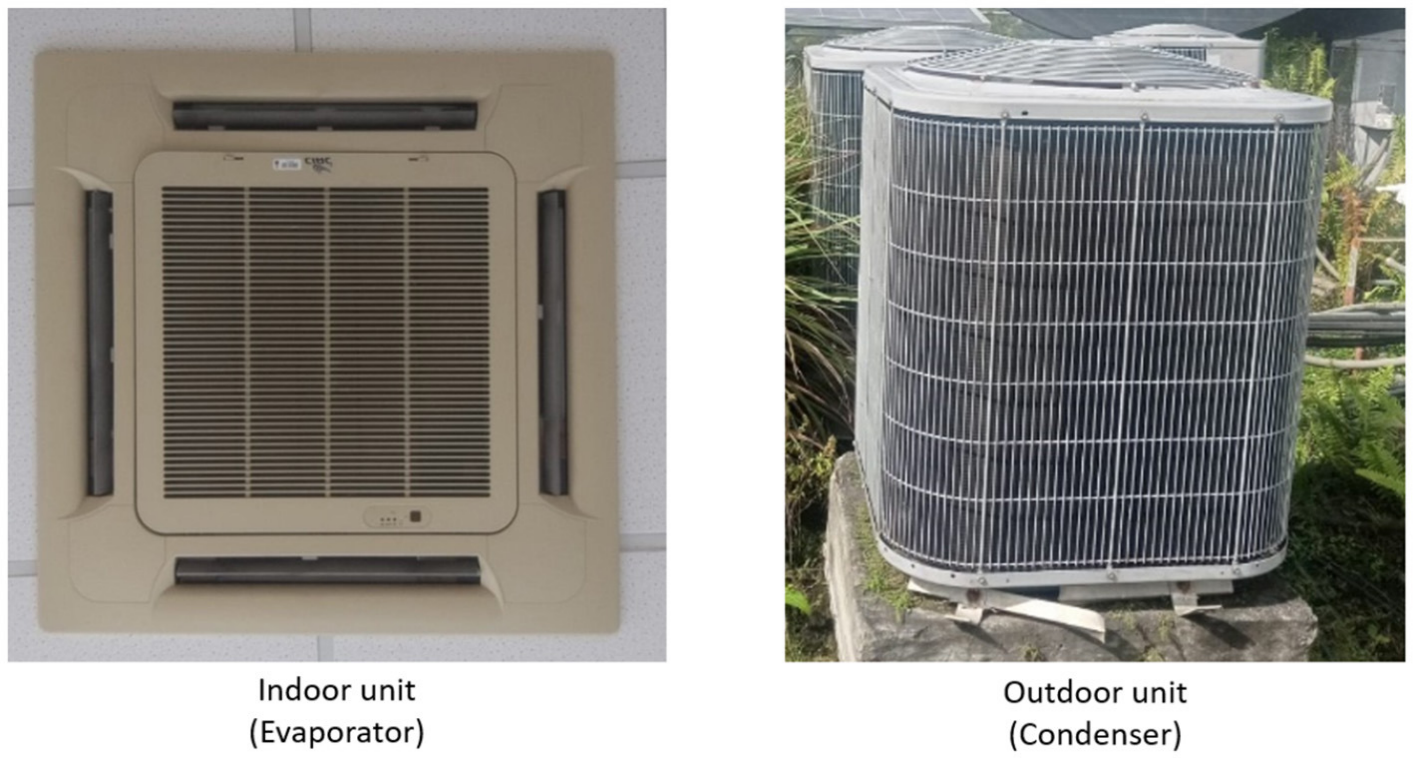
Classroom's air conditioning.

The case study of this research is a classroom in an educational building located in Bucaramanga, Colombia (Figure 1). This city has a tropical climate, which is warm, humid, and rainy throughout the year, with temperatures ranging between 18°C and 28°C (Underground, 2024). Bucaramanga is geographically located at latitude 7.1193° north and longitude 73.1227° west. The classroom is used for undergraduate and graduate classes, mainly in electrical engineering, electronics, and telecommunications. The classroom is located on the fourth floor of the building and is equipped with two 36,000 BTU/h fan coil cassette air conditioners (Figure 2), as well as cross and forced ventilation. It has an area of 77.18 *m*^2^, a capacity for 34 people, 12 luminaires of 80 W, two flood lamps of 28 W, a projector, television, computer, and two air extractors.

### 2.2 Data collection

**Figure 3:**
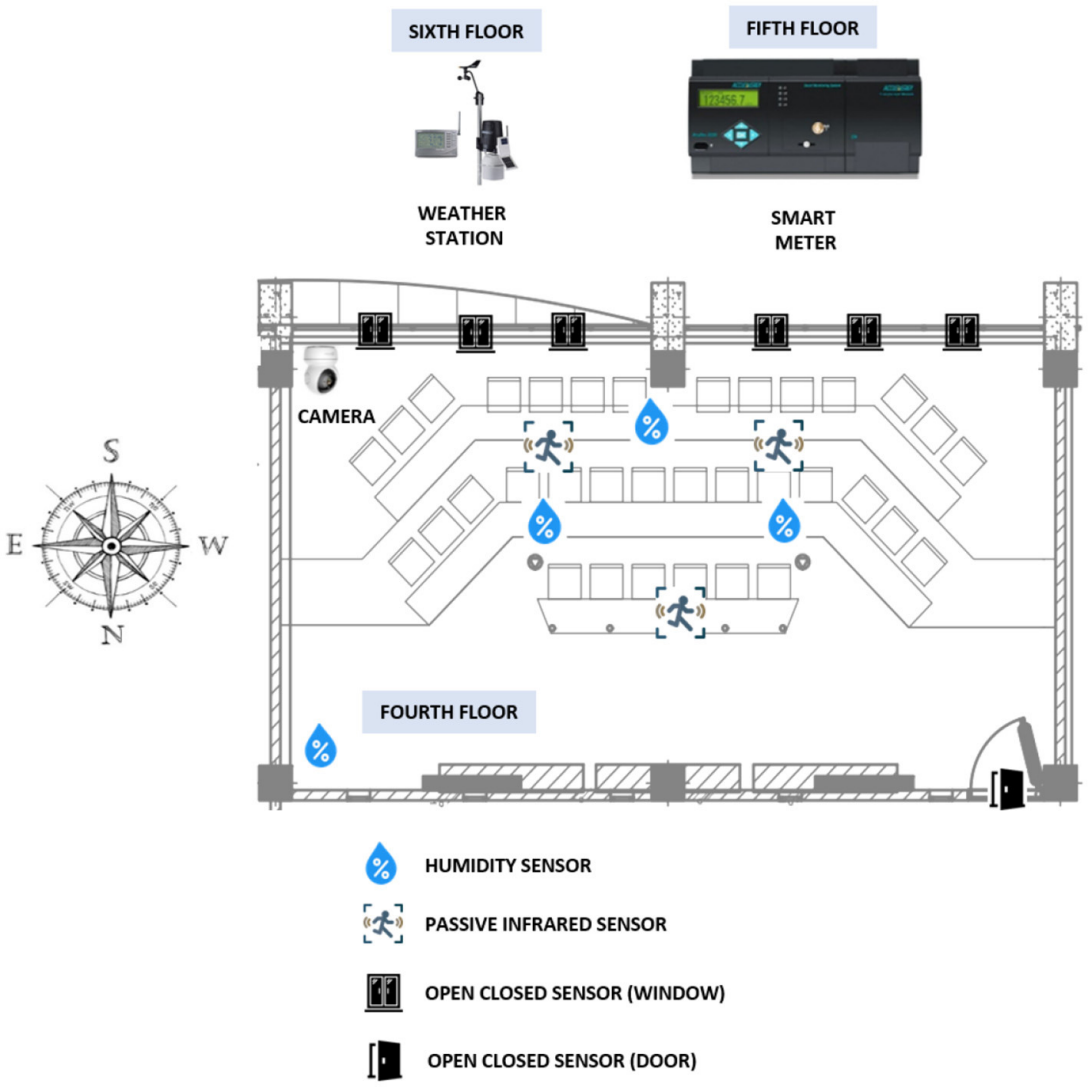
Classroom monitoring.

The data used in this research came from four primary sources: an energy meter, a weather station, wireless sensors, and a video camera (Figure 3). The sensors and camera were installed inside the classroom, while the weather station was located on the building's terrace on the sixth floor. The energy meter was located on the fifth floor, where the general low-voltage switchboard for the air conditioners is located. A detailed description of the equipment, the time stamps used for monitoring, and the selected variables can be found in Table 1. The monitoring was done for approximately two and a half months, from February 19 to April 30, 2024.

**Table 1:** Monitoring equipment, characteristics, sampling time, and monitored variables.

**Equipment**	**Characteristics**	**Time stamping**	**Variables**
ACUREV 2020	Multi-circuit power and energy meter Monitors 9 single-phase circuits external 333 mV CT input	5 min	Energy [Wh]
DAVIS VANTAGE PRO 2	Professional quality weather station Wireless wide range of sensors	5 min	Outdoor temperature [°C] Outdoor humidity [%] Dew point [°C] Wind speed [m/s] Wind direction (Categorical) Heat index [°C] Atmospheric pressure [mmHg] Rain rate [mm/hr] Solar radiation [W/m^2^] UV index cooling degree days [°C]
CAMERA	Smart home camera with motion detection and 1080p resolution two-way conversation.	5 min	Occupant number [#] Computer number [#]
MONNIT	Wireless sensors: humidity, open closed, and PIR	10 min	Indoor humidity [%] Indoor temperature [°C] Movement (Categorical) Opening and closing of door and windows (Categorical)

Variables that do not have units are categorical or have no units.

### 2.3 Data processing

**Figure 4:**
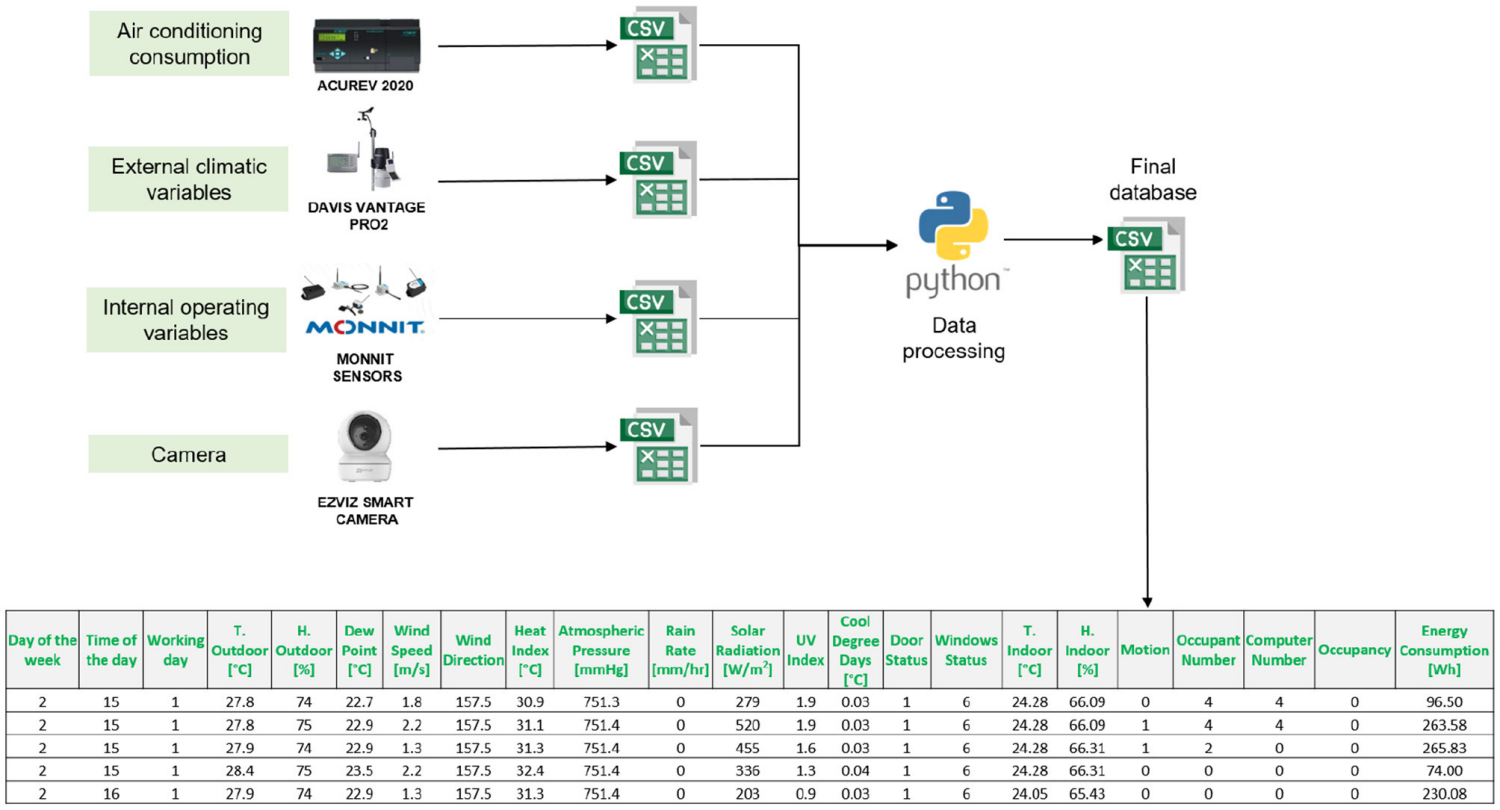
Data processing scheme.

For data processing, it was necessary to merge the data from each monitoring equipment to have a final database (Figure 4). Each equipment generates a comma-separated file (CSV) with the variables it monitors, except for the video camera, which provides the videos. The counting of people and computers was carried out manually from the videos. Using Python, a table is generated that integrates the most important variables: energy consumption of air conditioners, external climatic variables, internal or operational variables, the number of occupants, and computers. The Monnit sensors have a 10-min time stamp; it was necessary to interpolate the data to reduce the time stamp to 5 min. This interpolation ensures that all variables in the database have the same time stamp. Tables 2–4 show the statistical description of the data. The categorical variables in the dataset are explained in Table 5. Each numerical value represents a categorical or characteristic type of the variable.

**Table 2:** Data description 1.

	**Energy consumption**	**T. Outdoor**	**H. Outdoor**	**Dew point**	**Wind speed**	**Wind direction**	**Heat index**	**Atmospheric pressure**
Count	20,435	20,435	20,435	20,435	20,435	20,435	20,435	20,435
Mean	38.8	25.0	80.7	21.3	0.6	243.9	27.0	753.4
Std	108.8	2.7	8.8	1.5	0.8	106.1	3.7	1.6
Min	0.0	19.0	53.0	15.8	0.0	0.0	20.2	748.6
25%	0.0	22.9	74.0	20.3	0.0	157.5	23.9	752.3
50%	0.0	24.7	80.0	21.3	0.0	202.5	26.1	753.5
75%	0.0	26.9	87.0	22.5	0.9	360.0	29.4	754.5
Max	413.9	32.8	98.0	24.7	3.6	360.0	38.8	758.1

**Table 3:** Data description 2.

	**Rain rate**	**Solar radiation**	**UV index**	**Cool degree days**	**Door status**	**Windows status**	**T. Indoor**	**H. Indoor**
Count	20,435	20,435	20,435	20,435	20,435	20,435	20,435	20,435
Mean	0.0	191.9	1.4	0.0	0.9	5.8	25.7	64.8
Std	0.1	284.6	2.4	0.0	0.3	0.8	1.5	6.2
Min	0.0	0.0	0.0	0.0	0.0	0.0	20.5	39.4
25%	0.0	0.0	0.0	0.0	1.0	6.0	24.7	61.6
50%	0.0	1.0	0.0	0.0	1.0	6.0	25.9	65.7
75%	0.0	316.0	2.3	0.0	1.0	6.0	26.9	69.2
Max	5.1	1254.0	11.4	0.1	1.0	6.0	29.2	79.3

**Table 4:** Data description 3.

	**Motion**	**Occupant number**	**Computer number**	**Occupancy**	**Day of the week**	**Time of the day**	**Working day**
Count	20,435	20,435	20,435	20,435	20,435	20,435	20,435
Mean	0.1	3.1	0.4	0.2	2.91	11.52	0.71
Std	0.4	7.3	1.7	0.4	0.0	0.0	0.0
Min	0.0	0.0	0.0	0.0	1.0	5.0	0.0
25%	0.0	0.0	0.0	0.0	3.0	12.0	1.0
50%	0.0	0.0	0.0	0.0	5.0	18.0	1.0
75%	0.0	0.0	0.0	0.0	6.0	23.0	1.0
Max	1.0	32.0	21.0	1.0	2.0	6.9	0.45

**Table 5:** Values of categorical variables.

**Variable**	**Meaning**	**Value**
Day of the week	Monday to Sunday	0–6
Time of the day	0 hours to 23 hours	0–23
Working day	Not working day Working day	0 1
Wind direction	N NNE NE ENE E ESE SE SSE S SSW SW WSW W WNW NW NNW −−−	0 22.5 45 67.5 90 112.5 135 157.5 180 202.5 225 247.5 270292.5 315 337.5 360
Door status	Closed Open	1 0
Windows status	Number of windows closed	0–6
Motion	Motion No motion	1 0
Occupancy	Occupied Unoccupied	1 0

#### 2.3.1 Normalization

Before training the models, it is necessary to normalize the data to improve its integrity, avoid biases due to influential variables, and help the training process. It is decided to use StandardScaler, a normalization technique that standardizes the data by removing the mean and adjusting the scale so that the variance equals one. The standard value of a variable *x* is calculated as shown in Equation 1:


(1) $$\begin{array}{c} z=\frac{\left(x-u\right)}{s} \end{array}$$

The z-value measures how far a value *x* is from the mean *u* of a data set, expressed in terms of the standard deviation *s*. It indicates how many standard deviations a value is above or below the mean. If z > 0, the value is above the mean; if z < 0, the value is below the mean; and if z = 0, the value matches the mean exactly. *x* represents the specific value being analyzed, *u* is the average of all values in the data set, and the standard deviation *s* measures the dispersion or variability of the data relative to the mean.

### 2.4 Model training

The data normalization and model training process was conducted using the Python programming language. Various libraries were employed to facilitate the process, including sklearn, tensorflow, pandas, matplotlib, and others. Table 6 shows the Python libraries used for model training, their function, and the model used. The models presented below were selected because of their wide application in regression tasks. There is no definitive guide in the literature for choosing a specific model, as the choice depends on the case study and data characteristics. Each of these models has proven effective in different contexts, making them suitable for evaluating their performance in this work. Additionally, hyperparameter tuning was performed for each model to optimize its performance and ensure the best possible results for the specific dataset used.

**Table 6:** Description of the libraries used.

**Library**	**Function**	**Model used**
Matplotlib	Creation of static, animated, and interactive plots and visualizations.	show, figure, xticks, ylabel, xlabel, grid, subplots, plot, scatter, title, legend
Pandas	Data manipulation and analysis using structures like DataFrame and Series.	read_csv, Dataframe, to_datetime, date_range
Tensorflow	Creation and training of machine learning models and neural networks.	-
Sklearn	Implementation of machine learning algorithms and modeling tools.	model_selection, metrics, linear_model, svm, ensemble
Numpy	Manipulation of arrays and advanced mathematical operations.	sqrt, mean
Seaborn	Data visualization based on Matplotlib with statistical analysis support	-
Scikit-learn	Data visualization based on Matplotlib with statistical analysis support.	preprocessing, model_selection, linear_model, metrics, svm, ensemble, tree, datasets, model_selection

#### 2.4.1 Cross-validation

Cross-validation is a technique used to evaluate the overall performance of a model and its effectiveness on unseen data. The purpose is to avoid underfitting the training data by ensuring the model generalizes well to other data. The data were divided into training and testing sets in an 80:20 ratio. The type of cross-validation used was kFold, which divides the training data set into “k” parts or folds in a balanced manner and then multiple iterations of training and validation. We defined the value of k as 4, as we consider it appropriate given the limited size of the data set, which makes further subdivision difficult. In addition, this value maintains a low computational cost and allows us to achieve a reasonable balance between bias and variance.

#### 2.4.2 Support vector regressor (SVR)

SVR is a Support Vector Machine (SVM) used for regression tasks. This algorithm works as follows: for each vector of input parameters *X* and its corresponding output vector *Y*, SVR relates these vectors using Equation 3 (Walker et al., 2020).


(2) $$\begin{array}{c} Y=W\varphi \left(X\right)+b \end{array}$$

Where *W* is the vector of weights and *b* represents the bias. These depend on the selected kernel function, which quantifies the similarity of two observations. In this study, the radial basis function (RBF) kernel transforms data into a higher-dimensional space, facilitating linear separation. It measures similarity using the Euclidean distance between points, enabling algorithms to capture complex patterns more effectively (Hofmann et al., 2008).

#### 2.4.3 Decision tree (DT)

This algorithm is used for regression and classification. It is similar to a tree, containing decision nodes and leaf nodes. This structure is generated through recursive partitioning of the data by the algorithm based on the values of the input features. The partition data is mined using the specific partitioning criterion for each decision node, which corresponds to a feature. The leaf nodes, on the other hand, represent the class or expected value. Decision trees can detect complex non-linear correlations between the features and the target variable and are suitable for working with numerical and categorical features (Saeideh et al., 2024).

#### 2.4.4 Random forest regressor (RFR)

RFR (Random Forest Regressor) is a prediction model that groups multiple DTs trained by bagging and random variable selection. Although DT uses recursive partitioning, it is an unstable learner, as small changes in the data can completely alter the tree structure. RF solves this problem using multiple trees instead of a single tree, which reduces instability by combining predictions from diverse trees. This diversity helps to compensate for instability, as DT is unbiased and, on average, provides correct predictions. However, combining similar trees would not reduce instability (Wang et al., 2018).

### 2.5 Model evaluation

The models are evaluated through performance metrics, which measure the models' ability to make accurate predictions. They compare the values obtained with the actual or expected values and provide a quantitative value of that relationship. The metrics selected in this study are the coefficient of determination (*R*^2^), Mean Absolute Error (MAE), and Root Mean Square Error (RMSE). The coefficient *R*^2^ indicates the proportion of the variability in the dependent variable that the independent variables can explain. The MAE represents the average differences between the actual and predicted values. The RMSE measures the standard deviation of the prediction errors. Various performance metrics allow for evaluating the models from different perspectives, providing a comprehensive assessment, as each metric focuses on distinct aspects of the prediction error. For example, the *R*^2^ explains the percentage of data variability captured by the models, the MAE is easy to interpret and is robust to outliers, and the RMSE reflects the prediction errors in the units of the target variable and penalizes significant errors. The above metrics are defined as follows:


(3) $$\begin{array}{c} R^{2}=1-\frac{\sum_{i=1}^{n}{\left(y_{i}-{ŷ}_{i}\right)}^{2}}{\sum_{i=1}^{n}{\left(y_{i}-ȳ\right)}^{2}} \end{array}$$

(4) $$\begin{array}{c} MAE=\frac{1}{n}\overset{n}{\underset{i=1}{\sum }}|y_{i}-{ŷ}_{i}| \end{array}$$

(5) $$\begin{array}{c} RMSE=\sqrt{\frac{1}{n}\overset{n}{\underset{i=1}{\sum }}{\left(y_{i}-{ŷ}_{i}\right)}^{2}} \end{array}$$

Where *y*_*i*_ is the actual data, ŷ_*i*_ is the predicted values, ȳ is the average value of the dependent variable, and *n* is the number of observations. These errors were selected because they are the most commonly used in the literature to evaluate the performance of predictive models. Using multiple metrics allows a more complete picture of model performance, as each error provides different information on accuracy and generalizability.

### 2.6 Sensitivity analysis

Two sensitivity analyses are performed, the first consisting of varying the time interval of the data. We evaluate how the models perform with 1, 5, 10, 20, 30, and 60-min intervals. The intervals were selected to vary uniformly up to 1 h. Intervals over 60 min were not considered, which would significantly reduce the available data. Generally, the literature states that the maximum interval used is 1 h. This analysis uses the RMSE as the metric to compare the models. The metric to be reviewed is the *R*^2^, taking the entire data set (20,435 samples). The second sensitivity analysis is the variation in the proportions in which the test and training data are split. The splits considered are 50:50, 60:40, 70:30, 80:20, and 90:10. The model's accuracy is expected to decrease as the granularity of the data increases. Cross-validation is applied in both sensitivity analyses, dividing the data into 80% for training and 20% for testing.

## 3 Results and discussion

**Figure 5:**
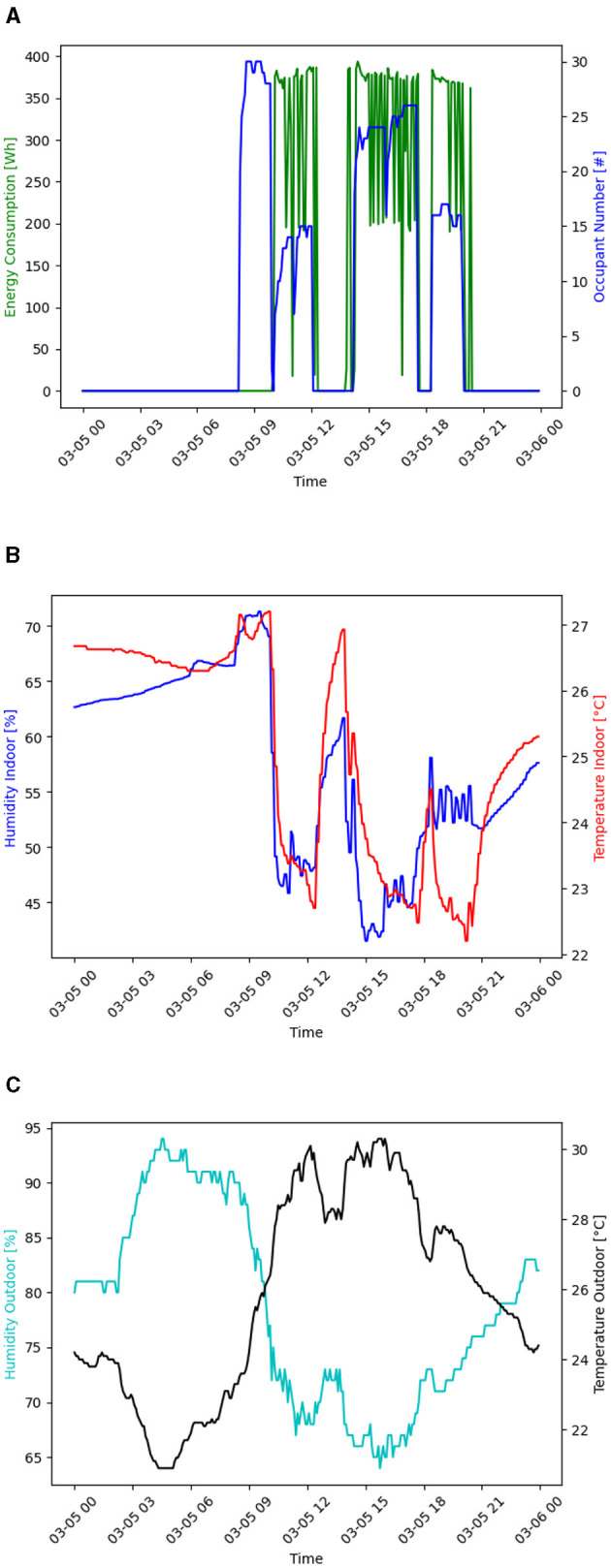
Behavior of some variables on March 5, 2024 **(A)** Energy consumption and occupancy behavior. **(B)** Indoor temperature and humidity behavior. **(C)** Outdoor temperature and humidity behavior.

Figure 5 presents the behavior of the number of occupants, energy consumption, indoor and outdoor temperature, and humidity on Tuesday, March 5, 2024. Tuesdays are the days with the highest energy consumption and flow of people. Figure 5A shows that classes begin at ~8:00 a.m.; however, no energy consumption is detected at that time due to the fresh climate characteristic of the city. At 10:00 a.m., although the number of people in the classroom decreases slightly, the air conditioners are turned on. There are no classes during midday between 12:00 p.m. and 2:00 p.m., so there is no occupancy or energy consumption. From 2:00 p.m. to 8:00 p.m., there is an increase in energy demand due to the use of the cooling system. In the first minutes of 6:00 p.m., there was a decrease in the occupancy and energy consumption values, corresponding to a change of class that left the space unoccupied. Figure 5B illustrates the same day's temperature and humidity dynamics inside the classroom. In the period from 8:00 a.m. to 10:00 a.m., there was an increase in both variables; the entry of people into the space and the turned-off state of the ACs were the leading causes of this increase. From 10:00 a.m. to 12:00 p.m., the indoor temperature and humidity decreased due to the turning on of the ACs, and the same happened from 2 to 8 p.m. At midday, the temperature and humidity increased significantly, reaching 27°C and 60% moisture, respectively. Figure 5C shows the behavior of the humidity and outside temperature, which have an inversely proportional relationship. At ~9 a.m., the outside temperature increases, coinciding with when the ACs are turned on in the classroom.

**Figure 6:**
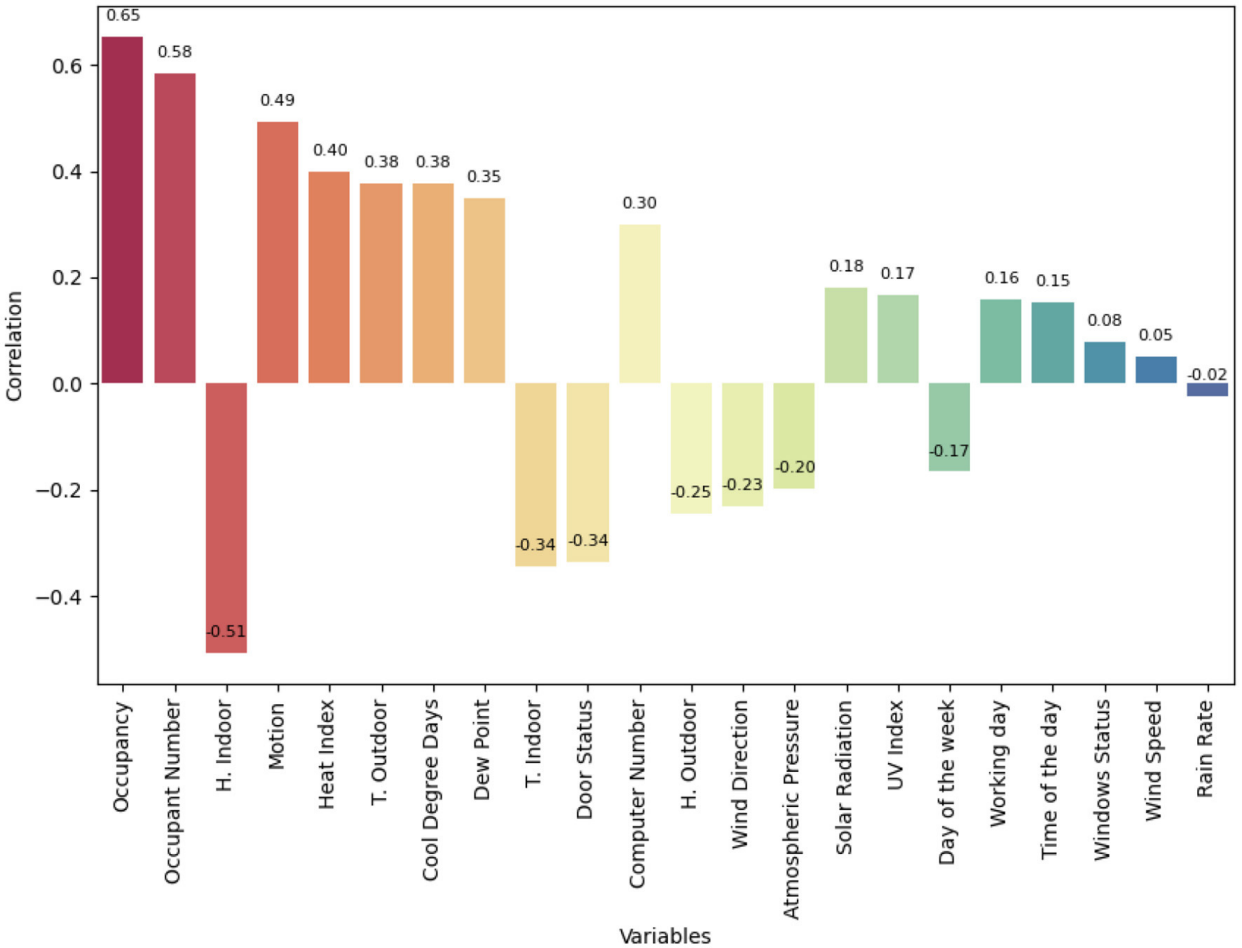
Correlation matrix.

Figure 6 shows the linear correlation plot between the variables in the data set with AC power consumption. The correlation describes the direction and strength of the relationship between the variables. In this case, the variables were ordered in descending order where occupancy, number of occupants, and indoor humidity correlate with the absolute value of 0.5. Indoor humidity has a negative correlation, as AC consumption increases, the humidity in the classroom decreases.

In the training, six data sweep scenarios were considered to evaluate the models' performance by varying the number of samples and monitoring period. These scenarios were selected based on the data from each monitored month. The vacation week was excluded, as no energy consumption was recorded during that period, which occurred in March 2024.

- **Scenario 1:** Take the complete dataset (20,435 samples)
- **Scenario 2:** Take the whole dataset and delete the vacation week (18,131 samples)
- **Scenario 3:** Take only February data (3,168 samples)
- **Scenario 4:** Take only March data (8,712 samples)
- **Scenario 5:** Take March data and remove data from the vacation week. (6,408 samples)
- **Scenario 6:** Take only April data (8,555 samples)

**Figure 7:**
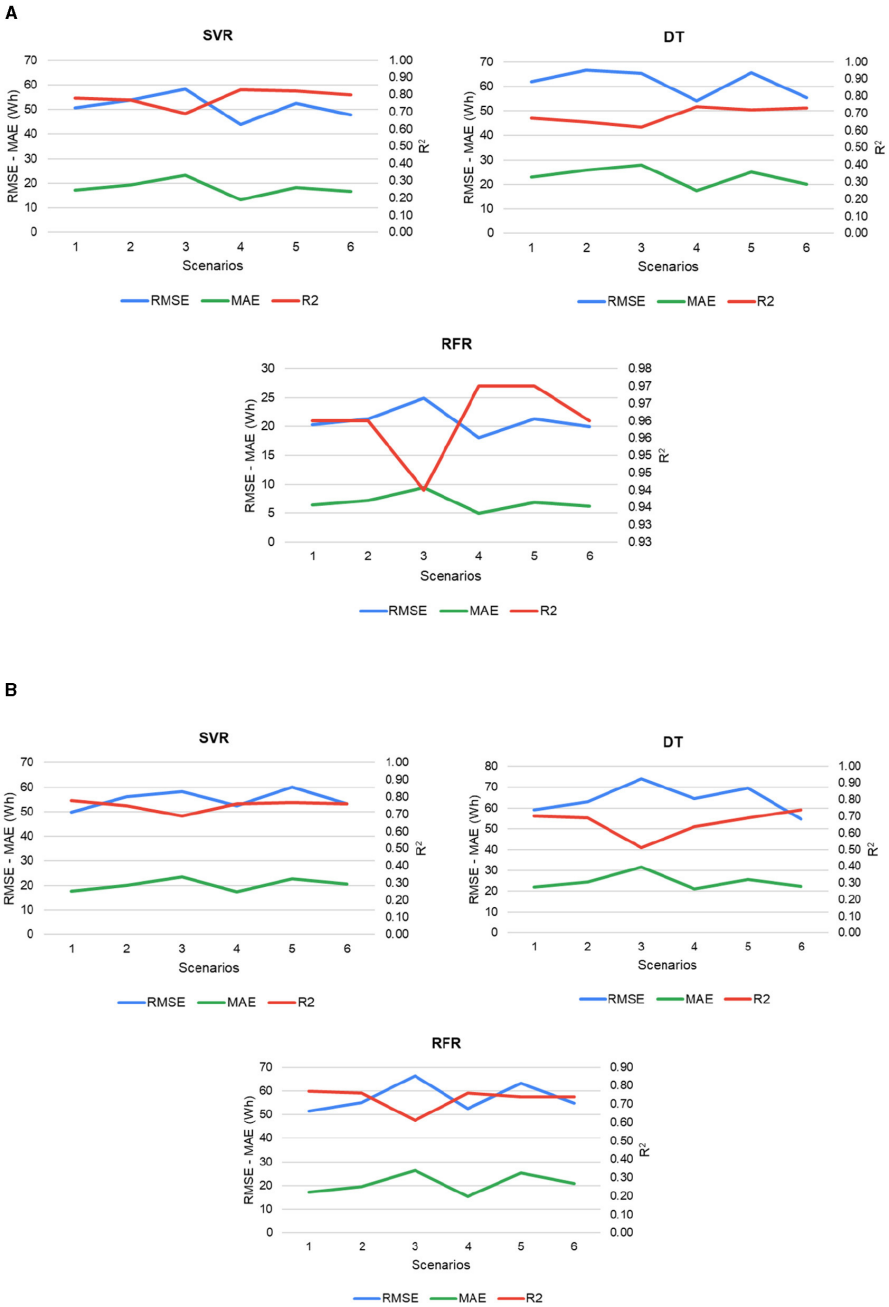
Errors at different stages of modeling **(A)** Model performance in the training process. **(B)** Final performance with test data.

Figure 7 illustrates the behavior of the models in each of the scenarios during the training and testing process. Figure 7A shows the performance of the models using 80% of the data corresponding to the training set. Cross-validation was used so that the values of the metrics are the average value of the results of each fold. For the three models evaluated, it is observed that the MAE, RMSE, and *R*^2^ present their best results in scenario 4. The best-performing model was the RFR, with an RMSE of 18.05 Wh, an MAE of 4.98 Wh, and an *R*^2^ of 0.97. Based on the above, the period corresponding to March presents consumption patterns and input variables that favor the best performance of the models compared to other scenarios. The process of testing with data not seen by the models is shown in Figure 7B, the highest *R*^2^ value was 0.78, and the lowest RMSE was 49.77 Wh with the SVR model in scenario 1. As for the MAE, the slightest error is 15.40 Wh obtained by the RFR model in scenario 4. From these results, it can be inferred that although a model may show excellent performance during training, its predictive ability may decrease when evaluated with new data. Such is the case of the RFR, which learns very well with training data but significantly decreases its predictive ability with test data. In addition, scenario 1, which did not yield the best metrics during training, allowed the models to learn more valuable patterns and maintain their range of error values.

**Figure 8:**
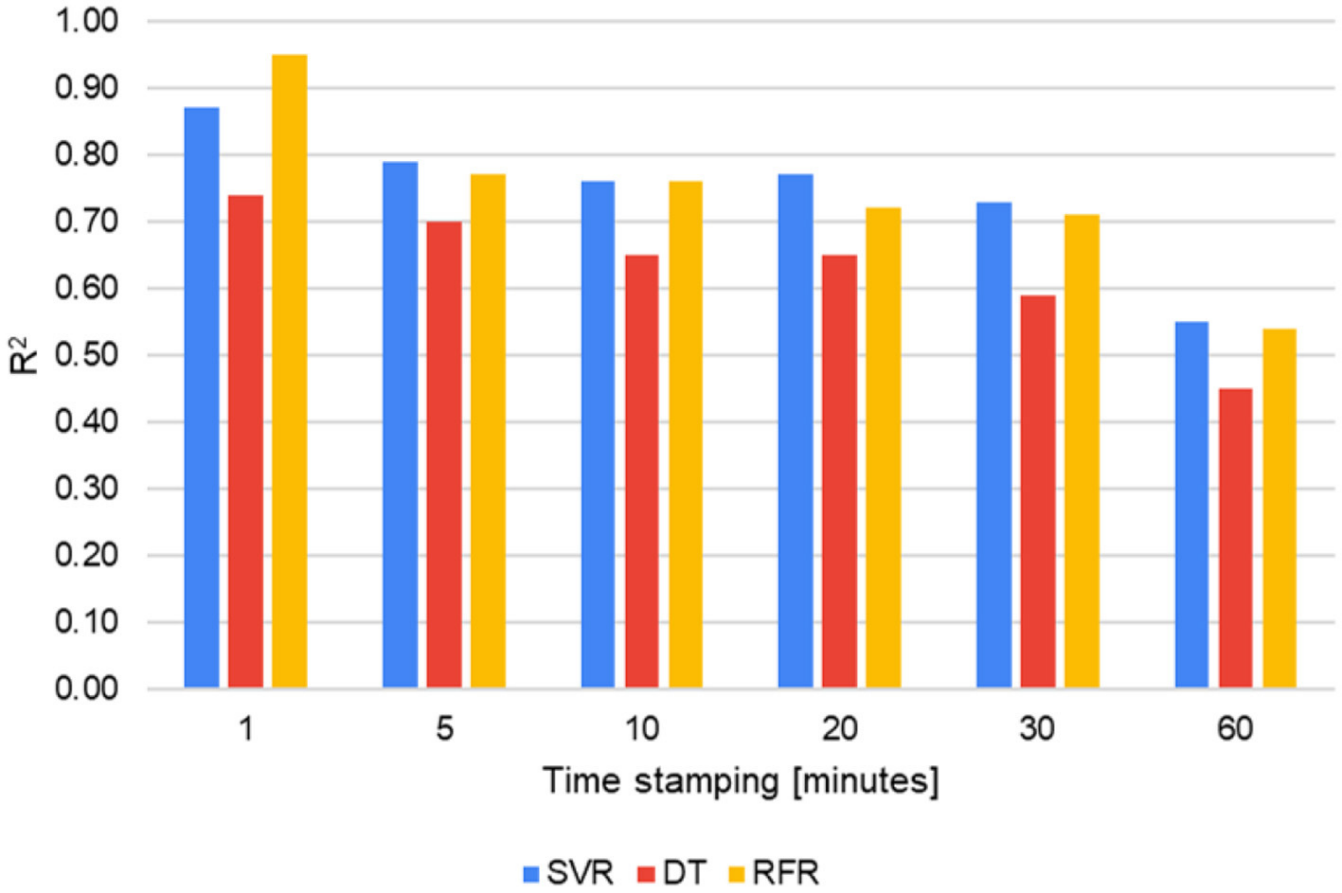
Performance of models for different time stamps.

The performance of the models is evaluated by varying the time stamping (Figure 8). The results shown correspond to the evaluation of the test data. Through *R*^2^, it is possible to pinpoint the effects of decreasing the time stamping of the data. The SVR and RFR models were above DT for all stamping types. RFR obtained an *R*^2^ of 0.95 with 1-min stamping (103,676 samples), the highest of all combinations. Increasing the time stamping or, in other words, decreasing the number of samples reduces the model's ability to generalize and adequately predict the energy consumption for cooling in the classroom.

**Figure 9:**
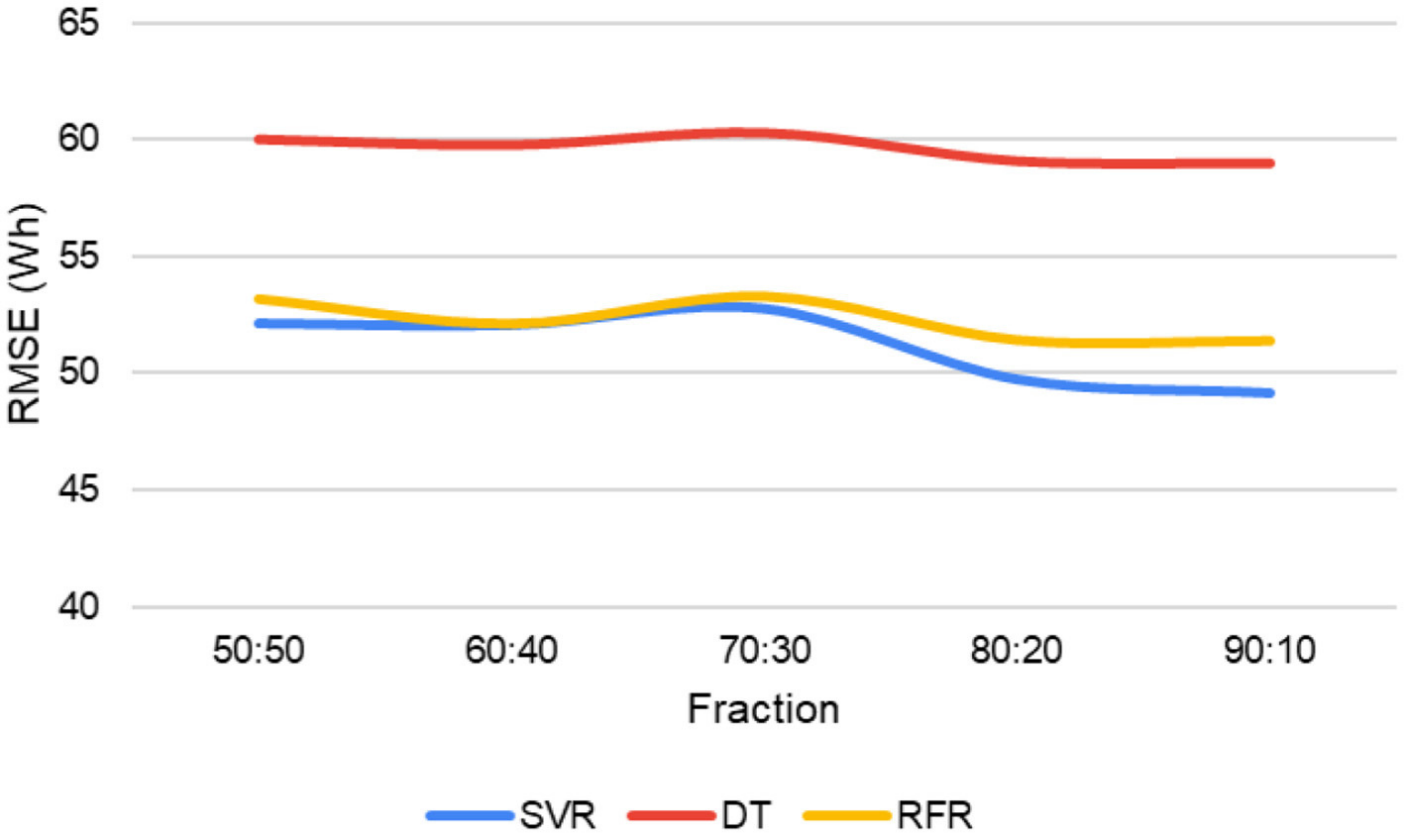
Performance of models for different data separation.

Figure 9 explains the behavior of the RMSE when modifying the data split at the training stage (cross-validation). The data split has the form a:b, where a is the percentage of training data and b is the percentage of validation data. The DT model performs less well than SVR and RFR for all data fractions. The errors of the other two models vary between 49 and 52 Wh, and they present some fluctuations where the 70:30 fraction has the highest RMSE value. The 90:10 fraction of the SVR model is the type of data separation that provides the lowest error. Thus, it can be deduced that the smaller the amount of test data and the larger the proportion of training data, the more clearly models can learn patterns from the models. The model is expected to know better with a more significant amount of data used for training. Sensitivity analyses were performed by evaluating the three errors considered in this study. However, the *R*^2^ for time stamping variation and the RMSE for data splitting best reflect the behavior of the models.

This study does not intend to generalize the energy consumption prediction in buildings but is a starting point for future research. In particular, it focuses on a specific case: the energy consumption of AC systems in classrooms. There is still a need for studies that address total building consumption and comprehensively consider factors such as climate, space use, occupancy, geographic location of the building, electronic equipment used, and type of construction, among others. In addition, a 72-day monitoring period is insufficient to generalize the results or develop a highly accurate model. More exhaustive and prolonged monitoring is required to more accurately interpret the energy consumption behavior of the AC systems in these spaces. This methodology could be extended to other classrooms, different areas of a building, or even entire buildings, provided that the same equipment is installed and variables are monitored consistently, facilitating comparison of results.

## 4 Conclusions

The variables that demonstrate a significant correlation with energy consumption for refrigeration in the classroom are occupancy, the number of occupants, indoor humidity, and movement. Tuesdays are the days of the week when most classes are taught in the classroom. Selected on March 5, 2024, it is evident that the dynamics of the use of the AC are related to the occupation, use of space, and the city's climate. Occupancy influences energy consumption due to the use of electronic equipment, lighting, and air conditioning. The use of space is related to occupancy schedules and is directly related to energy consumption. Climate is also a determining factor: in tropical climates, air conditioning is constant, while in temperate climates, where there are seasons, energy consumption varies according to the time of year, alternating between heating and cooling. In addition, cultural factors also influence energy consumption patterns since occupants' practices and preferences can directly affect the use of air conditioning and lighting systems. In the case of the data sweep scenarios, it was found that the RFR model presents an excellent level of generalization with the training data; however, in the testing stage, the SVR model performs more accurate predictions. Finally, the trend in the prediction of the models is that the *R*^2^ decreases as the time stamping of the data decreases. Because of the above, a data set with reduced time intervals close to 1 min is recommended. Considering 80% or more of the data for training is a good practice when looking for models to learn complex patterns, especially with large, information-rich datasets. Better generalization capabilities are often achieved if the validation set adequately reflects the variability of the data. The results of this study provide a basis for future research aimed at improving energy consumption prediction models. These models can be integrated into building energy management systems, such as BAS (Building Automation System), BEMS (Building Energy Management System), or digital twins. Implementing these systems allows for more efficient energy consumption management, decision-making, preventive and corrective measures, and timely maintenance. In addition, building managers can invest in these systems and recover the investment through optimization and efficient energy use.

## Data Availability

The raw data supporting the conclusions of this article will be made available by the authors, without undue reservation.
